# Emoji Education: How Students Can Help Increase Health Awareness by Making Emojis

**DOI:** 10.2196/39059

**Published:** 2022-11-11

**Authors:** Sammer Marzouk, Shuhan He, Jarone Lee

**Affiliations:** 1 Department of Chemistry and Chemical Biology Harvard College Cambridge, MA United States; 2 Massachusetts General Hospital Boston, MA United States

**Keywords:** emoji, medical education, technology, education, medical students, creativity, student, health awareness, health, awareness, medical, society, innovation, communication, medical communication, electronic, artistic, representation

## Abstract

Emojis can improve health communication, especially when incorporating emojis into traditionally word-only texts. Beyond improving communication, emojis also offer greater access to health care, especially for vulnerable and marginalized populations with limited health literacy. A recent study found that 94% of patients with limited health literacy preferred health reports with emojis. Moreover, health officials are considering adding emojis to cardiopulmonary resuscitation guidelines and public health guidelines for handwashing. As the world evolves with new technology and new methods of communication, we must also evolve the language and method we use to communicate health information to patients. In this viewpoint, we aim to discuss the methods health care professionals can use to develop novel communication methods using emojis and the benefits of their incorporation into health care communication.

Emojis can improve health communication, especially when incorporating emojis into traditionally word-only texts [1]. Beyond improving communication, emojis also offer greater access to health care, especially for vulnerable and marginalized populations with limited health literacy. A recent study found that 94% of patients with limited health literacy preferred health reports with emojis [2]. Moreover, health officials are considering adding emojis to cardiopulmonary resuscitation (CPR) guidelines [3] as well as public health guidelines for handwashing [4]. As the world evolves with new technology and new methods of communication, we must also evolve the language and method we use to communicate health information to patients. In this viewpoint, we aim to discuss the methods health care professionals can use to develop novel communication methods using emojis and the benefits of their incorporation into health care communication.

A major obstacle for increasing the use of emojis in health care is the lack of diversity in health care–related emojis. Of the 3521 emojis in the Unicode Standard, which is the organization that maintains text and communication standards across electronic devices, only 30 of them were related to health care [5]. These mainly were generic body parts (eg, ears, hands, or legs) or general symbols such as “pill” or “syringe.” There is a large deficiency of emojis that communicate detailed aspects of health care, such as CPR, drawing blood, and getting an injection [6]. As such, we believe that the current generation of upcoming medical students have the potential to fill this gap with new emojis.

As members of a new generation that sends over 10 billion emojis a day, we have the greatest experience in knowing how to balance the artistic features of an emoji with the necessary detail to convey the information correctly. Researchers have proposed the development of new health-related emojis such as a liver emoji [7] and kidney emoji [8]. However, tech-savvy medical students have the ability to increase the use and accessibility of emojis by just increasing the use of emojis in common health media. Using common applications such as *inTextMoji*, *Bitmoji*, *Avatoon*, and many more, we can place colorful, creative, and inviting representations onto traditionally text-heavy guides in medicine. Most applications for emoji-making involve converting pictures or drawings into Unicode pictures that you can send through text, so students can use their talents in digital arts to establish new symbols and representations [9]. Applications such as *EmojiRequest* allow users to submit their designed emojis to a public contest, where the most popular emojis become publicly available on their phone app and website. Eventually, popularly requested emojis will be proposed to the Unicode Standard—acceptance into which will cause these emojis to be available on handheld devices worldwide.

Anyone can submit an application for a new emoji design to the Unicode Standard. There is a submission window from April 4 to July 31 every year for unique emoji designs. For the emoji application, a new design has to have a descriptive name, a category that it fits into, and a reason that necessitates its inclusion into the standard. For making decisions, the Unicode Consortium focuses on if novel emojis fit its selection factors, which are its metrics for deciding if there is a public need for the novel emoji design. These factors include its distinctiveness from other emojis, its expected use levels based on internet search analytics from similar topics, and the cultural universality of the emoji design [10]. The most important metric is the expected use levels, which is demonstrated by comparing the search popularity of the emoji’s topic with the term *elephant*. The emoji’s topic will be compared to *elephant* using Google Trends, Bing Trends, and general search analytics to show there is a public interest in the topic depicted in the emoji. This is because the *elephant* emoji is not the most popular or unpopular emoji, so it will be an indicator of the potential popularity of the novel emoji. Potential emojis must be submitted in a 18 × 18 pixel size, which is the size of emojis in a phone, and a 72 × 72 pixel size.

After initial submission, applicants will wait 2 to 6 weeks to see if their emoji has passed the basic review and if it would be presented to the Unicode Technical Committee (UTC) for full consideration. Emojis that do not make it past this stage cannot be reconsidered for 2 years. At the UTC meeting, around 50 to 70 new emojis will be discussed [11], and the final emoji list will be released around early March of the following year. It will usually take several months for emoji vendors such as Apple, Twitter, and Google to approve new emojis from the UTC and release them on their platforms.

Even though it might be a lofty goal, previous grassroots-based movements have successfully advocated for new emojis to be added to the Unicode Standard based on popular request. Most notably this was the development of a wide array of skin colors for emojis to allow for greater representation of different peoples. With using health care emojis, we are able to increase the accessibility of health care information to a larger audience. Emojis have been used in diverse patient populations such as older adult patients, non-English–speaking patients, and young children [12,13]. All of these groups reported having an increased understanding of the health care information being conveyed through emojis rather than through traditional communication methods [5,14,15]. We want the readers to use their creativity and experience to kick-start the future and much needed evolution of health care communication.

## Abbreviations

CPR: cardiopulmonary resuscitation
UTC: Unicode Technical Committee
